# Microstructural, Electrical, and Tribomechanical Properties of Mo-W-C Nanocomposite Films

**DOI:** 10.3390/nano14121061

**Published:** 2024-06-20

**Authors:** Kateryna Smyrnova, Volodymyr I. Ivashchenko, Martin Sahul, Ľubomír Čaplovič, Petro Skrynskyi, Andrii Kozak, Piotr Konarski, Tomasz N. Koltunowicz, Piotr Galaszkiewicz, Vitalii Bondariev, Pawel Zukowski, Piotr Budzynski, Svitlana Borba-Pogrebnjak, Mariusz Kamiński, Lucia Bónová, Vyacheslav Beresnev, Alexander Pogrebnjak

**Affiliations:** 1 Faculty of Materials Science and Technology in Trnava, Slovak University of Technology in Bratislava, 25, J. Bottu St., 917 24 Trnava, Slovakia; ivashchenko@icnanotox.org (V.I.I.); martin.sahul@stuba.sk (M.S.); lubomir.caplovic@stuba.sk (Ľ.Č.); lucia.bonova@stuba.sk (L.B.); 2 Biomedical Research Centre, Sumy State University, 116, Kharkivska St., 40007 Sumy, Ukraine; 3 Frantsevich Institute for Problems of Materials Science, National Academy of Sciences of Ukraine, 3, Krzhizhanovsky St., 03142 Kyiv, Ukraine; petro@ipms.kiev.ua; 4 Institute of Electrical Engineering, Slovak Academy of Sciences, 9, Dúbravská Cesta St., 841 04 Bratislava, Slovakia; andrii.kozak@savba.sk; 5 Łukasiewicz Research Network—Tele and Radio Research Institute, 11, Ratuszowa St., 03-450 Warszawa, Poland; piotr.konarski@itr.lukasiewicz.gov.pl; 6 Faculty of Electrical Engineering and Computer Science, Lublin University of Technology, 38A, Nadbystrzycka St., 20-618 Lublin, Poland; t.koltunowicz@pollub.pl (T.N.K.); p.galaszkiewicz@pollub.pl (P.G.); v.bondariev@pollub.pl (V.B.); 7 Department of Economics, Vincent Pol University in Lublin, 2, Choiny St., 20-816 Lublin, Poland; zukowski50pawel@gmail.com; 8 Faculty of Mechanical Engineering, Lublin University of Technology, 36, Nadbystrzycka St., 20-618 Lublin, Poland; p.budzynski@pollub.pl (P.B.); mariusz.kaminski@pollub.pl (M.K.); 9 Faculty of Electronics and Information Technology, Sumy State University, 2, Rymskogo-Korsakova St., 40007 Sumy, Ukraine; borbac@i.ua; 10 Department of Reactor Engineering Materials and Physical Technologies, V.N. Karazin Kharkiv National University, 4, Svobody Sq., 61022 Kharkiv, Ukraine; v.beresnev@karazin.ua

**Keywords:** electrical properties, conductivity, nanocomposites, refractory metal carbide, wear resistance, sliding friction

## Abstract

This study investigates the phase composition, microstructure, and their influence on the properties of Mo-W-C nanocomposite films deposited by dual-source magnetron sputtering. The synthesised films consist of metal carbide nanograins embedded in an amorphous carbon matrix. It has been found that nanograins are composed of the hexagonal β-(Mo_2_ + W_2_)C phase at a low carbon source power. An increase in the power results in the change in the structure of the carbide nanoparticles from a single-phase to a mixture of the β-(Mo_2_ + W_2_)C and NaCl-type α-(Mo + W)C_(0.65≤*k*≤1)_ solid-solution phases. The analysis of electrical properties demonstrates that the nanograin structure of the films favours the occurrence of hopping conductivity. The double-phase structure leads to a twofold increase in the relaxation time compared to the single-phase one. Films with both types of nanograin structures exhibit tunnelling conductance without the need for thermal activation. The average distance between the potential wells produced by the carbide nanograins in nanocomposite films is approximately 3.4 ± 0.2 nm. A study of tribomechanical properties showed that Mo-W-C films composed of a mixture of the β-(Mo_2_ + W_2_)C and α-(Mo + W)C_(0.65≤*k*≤1)_ phases have the highest hardness (19–22 GPa) and the lowest friction coefficient (0.15–0.24) and wear volume (0.00302–0.00381 mm^2^). Such a combination of electrical and tribomechanical properties demonstrates the suitability of Mo-W-C nanocomposite films for various micromechanical devices and power electronics.

## 1. Introduction

Studies on carbide ceramic materials with various additives show that these materials have numerous applications across different fields. Various transition metal carbides can be effective in tumour treatment and tissue engineering [1,2,3]. Molybdenum carbide alloyed with tungsten with a high melting point is widely used in mechanical engineering as a refractor additive for cutting tools. For instance, the coating of MoC and Mo_2_C carbides deposited by arc vacuum physical vapour deposition on the HS 18-0-2-5 steel blades of milling tool knives demonstrated an increase in their hardness and wear resistance compared to the bare tools [4,5]. The Mo_2_C in the form of a fine-scale dispersion in steel and ceramic materials is an excellent reinforcement phase, contributing to a secondary hardening behaviour [6]. The MoC is also utilised as an integral part of a highly thermally conductive microwave energy absorber. In addition, MoC is a superconductor already at a temperature of 9.26 K, and in another phase, Mo_2_C, this temperature is even lower, about 2.7 K [7,8,9,10]. 

Many papers have demonstrated that doping films and coatings with various metals can enhance their functional properties [11,12]. Adding a third element can improve the overall performance of a material by tailoring different parameters such as the lattice constant, preferred orientation, grain size, stresses, coefficient of thermal expansion, etc. In particular, W has been found to be effective in improving the mechanical and tribological properties of a wide range of materials. For instance, adding tungsten to CrN coatings increased covalent bonding, enhancing hardness, adhesion strength, oxidation resistance, and reducing residual stresses [13]. Doping of the microarc oxidation coating on a Ti_6_Al_4_V drill pipe with W also improved the hardness and corrosion resistance of the base coatings [14]. Friction tests of W-doped diamond-like carbon coatings lubricated by molybdenum dithiocarbamate also demonstrated a positive effect of tungsten on reducing the friction coefficient [15].

The recent studies of nanostructured carbon-based coatings doped with Mo and W (Mo-W-C) revealed their high thermal stability up to ~800 °C and excellent tribological properties at different temperatures [16,17,18]. Moreover, Page et al. [19] demonstrated that nanostructured Mo_2_C and WC with a large surface area can be used for catalytic processes as substitute materials for platinum group metal heterogeneous catalysts. In one of the recent works, Mo-W-C coatings with three different C/(Mo + W) ratios (5:1, 2.8:1, 2.2:1) were synthesised using unbalanced magnetron sputtering combined with high-power impulse magnetron sputtering [20]. The research results showed that coatings with low values of the carbon-to-metal elements ratio had the best tribological properties during lubricated sliding. Enhanced properties were related to the formation during the friction at 200 °C with lubricant containing anti-wear and sulfide-based additives MoS_2_ and WS_2_ due to chemical reactions and the presence of amorphous carbon in the tribolayer. As the sliding distance increased, the tribolayer reduced the coefficient of friction and protected the coating from severe wear. However, samples with a high C/(Mo + W) ratio of about 5:1 were characterised by significant abrasive wear. While Mo-W-C films show promising characteristics, their microstructure features and properties are not yet fully understood.

This paper discusses the Mo-W-C nanocomposite films synthesised by a dual-source magnetron sputtering method. The effect of carbon source power on chemical composition, phase formation, hardness, tribological behaviour, conductivity, and dielectric permittivity of deposited nanocomposites was comprehensively studied.

## 2. Materials and Methods

### 2.1. Deposition of nc-MoWC + α-C Nanocomposite Films

The Mo-W-C films were deposited by DC dual magnetron sputtering (Frantsevich Institute for Problems of Materials Science of NASU, Kyiv, Ukraine). The scheme of the dual magnetron sputtering device was presented in papers [21,22]. Two disc-shaped targets with a diameter of 72 mm and a thickness of 4 mm were used: Mo_0.85_ W_0.15_ alloy and graphite (C) (Frantsevich Institute for Problems of Materials Science of NASU, Kyiv, Ukraine). They were installed with an angle of 86° between them. The distance from the centre of each cathode was 90 mm. The polished Si (100) wafers were used as substrates. They were cleaned in the bath with a 5% HF solution, rinsed in de-ionised water, and dried in nitrogen. Before deposition, the substrates were etched in argon plasma in the reaction chamber. The vacuum chamber was first evacuated to a residual pressure of about 10^−3^ Pa. After adding the Ar gas to the chamber, the working pressure during the deposition of films was kept at about 0.2 Pa. The deposition parameters are listed in Table 1. The power supplied to the Mo-W target was fixed at approximately 66 W, whereas power on the graphite target, *P_C_*, varied in the range of 15–110 W. Hence, five Mo-W-C films were synthesised. The bias voltage applied to the substrates was set at −50 V. Furthermore, prior to the deposition, they were heated to 400 °C for better adhesion. The total deposition time was about 60 min.

### 2.2. Film Characterisation

The phase composition was studied by Grazing incidence X-ray diffractometry (GI-XRD) using a Panalytical Empyrean X-ray diffractometer (Malvern Panalytical, Malvern, UK) (U = 40 kV and I = 40 mA, ω = 1°). The Co-Kα radiation source with *λ* = 1.78901 Å was employed. The data were collected within the 2Theta interval of 30–100°. The step size was 0.053. An incident beam path consisted of a parallel beam mirror with a fixed 1/16° programmable divergence slit, a 0.04 rad Soller slit, and a 10 mm fixed mask. The crystallite sizes, *L*, were estimated by the modified Scherrer equation [23] and the Williamson–Hall method [24]. The crystallite size was calculated as an average value based on these two methods. The modified Scherrer equation can be given as:

(1) $$\ln\beta =\ln\frac{K\lambda }{L\cos\theta }=\ln\frac{K\lambda }{L}+\ln\frac{1}{\cos\theta },$$

where *β*—full width at the half maximum (FWHM) of peaks (in radians), *K*—shape constant with a value of 0.9, *L*—crystallite size, *λ*—wavelength of the X-ray, and *θ*—Bragg angle (in radians).

Plotting the dependence of ln*β* on ln(1/cos(*θ*)), one can obtain an intercept. The crystallite size can be found as an exponential of the intercept as follows:

(2) $$e^{\ln\frac{K\lambda }{L}}=\frac{K\lambda }{L}.$$

The Williamson–Hall method suggests that crystallite size (*L*) and strain (*ε*) can be found using the following equation:

(3) $$\beta \cos\theta =\frac{K\lambda }{L}+4\varepsilon \sin\theta .$$

If we plot 4*ε*sin(*θ*) on the x-axis and *β*cos(*θ*) on the y-axis, the y-intercept is used to find a crystallite size, and a slope of the linear fit is a strain component.

The morphology and chemical composition of the deposited films were investigated by a 7600F high-resolution field emission scanning electron microscope (SEM) JEOL JSM (Tokyo, Japan) equipped with an energy-dispersive X-ray spectroscopy detector (EDS) X-max 50 mm^2^.

Secondary-ion mass spectrometry (SIMS) analysis was conducted for the S4 sample using a 5 keV O^2+^ ion beam to study the element depth profiling. Hiden SIMS Workstation (Hiden Analytical, Warrington, UK) was equipped with an IG20 ion gun and a Maxim HAL7 quadrupole mass analyser (9 mm diameter rods). Prior to depth profile analysis, the mass spectrum was registered at the following conditions: the O_2_^+^ ion beam current and energy were 250 nA and 5 keV; the emission current was 8 mA; analysed masses of positive ions: ^12^C, ^16^O, ^30^Si, ^94^Mo, ^182^W; raster area was 500 µm × 600 µm; the electronic gate was set at 1%, 3%, and 10%. In order to quantify the SIMS data, the EDS results for sample S4 were used. Then, to perform a quantitative analysis, the ion ratios of given ion currents IX to the sum of the ion currents of all measured elements were calculated. To determine the sensitivity coefficients *k_X_* for individual element *X*, we divide the bulk EDX concentration c_X_ of element X by the secondary ion current I_X_ of this element. Ion current ratio (*IR_X_*), i.e., concentration, was defined for all elements detected in the film (C, O, Si, Mo, and W). The formula for the ion ratios was as follows: *IR_X_* = *I_X_k_X_*/(*I_C_k_C_* + *I_O_k_O_* + *I_Si_k_Si_* + *I_Mo_k_Mo_* + *I_W_k_W_*). The *k_X_* is a sensitivity coefficient for element *X*, which can be found as the bulk EDS concentration of element X divided by the secondary ion current *I_X_* of this element. The sputtering depth was calibrated by stylus profilometry using Tencor alpha-step 100 (KLA-Tencor, Milpitas, CA, USA). The resulting thickness of the deposited S4 sample was about 670 nm. Assuming the constant sputtering rate of the film, we converted sputtering time into depth.

The XPS analyses were carried out in a UHV chamber (10^−10^ Pa) of a photoelectron spectrometer (Prevac EA15, Zurich, Switzerland) equipped with a focused X-ray source (Al Kα 1486 eV). Prior to the analysis, etching with an Argon ion source was carried out at 2 keV for 30 min. The collected XPS data were analysed using CasaXPS 2.3.26.

Measurements of the electrical parameters of Mo-W-C films were studied using a test stand designed at the Department of Electrical Devices and High Voltage Technology of the Lublin University of Technology, Poland. The details of the procedure can be found in [25]. The CS 204AE-FMX-1AL helium cryostat (Advanced Research Systems, Inc., Macungie, PA, USA) was employed to perform measurements within the temperature range from 20 K to 375 K with an accuracy of 0.002 K. A vacuum of about 0.2 atm was used. The voltage amplitude supplied to the film was 0.4 V. Silver paste was applied as a thin layer at both ends of the sample to minimise the transition resistance at the junction between the sample and the contacts.

The Knoop hardness was determined using a Micromet 2103 (Blueher Ltd., Lake Bluff, IL, USA) microhardness tester at a load of 100 mN. Such condition of indentation was chosen to avoid an influence of the substrate material (the depths of the indents were no more than 9–10% of film thicknesses). Ten measurements were performed for each sample.

Tribological tests were carried out to measure the coefficient of friction and wear loss via the pin/ball-on-disk method using an Anton Paar nanotribometer (NTR2) (Graz, Austria) under dry friction conditions and at room temperature. One cycle corresponded to one rotation of the sample. The friction radius was 2.0 mm, and the rotational speed of the sample was 60 rpm (linear velocity at the friction node was 1.26 cm/s). A 0.5 mm diameter WC ball was used as a countersample during sliding. The applied load was 0.75 N. Wear was measured as the cross-sectional area of the wear track on the surface of the sample. For each wear track, 20 evenly spaced profilograms were taken, and then the average wear value was calculated. Measurements were taken using a Taylor–Hobson Form Talysurf Intra profilometer (Taylor Hobson, Leicester, UK) equipped with a measuring needle with a sphere tip with a rounding radius of 2 µm.

The Raman spectra at ambient conditions were measured by Raman microscope Alpha 300R (WiTec, Ulm, Germany) with an excitation wavelength of 532 nm. The power of the laser source was 1 mW. To interpret the Raman spectra of the deposited coatings, we used the results of the calculations of the special quasi-random structures and the phonon densities of states (PHDOSs) of cubic sub-stoichiometric α-MoC*_k_* (space group Fm-3m, No. 225) obtained in our previous paper [26]. Here, we calculated the PHDOSs of cubic WC_0.5_ and the hexagonal high-temperature β-Mo_2_C phase (space group P63/mmc, No. 194). The phonon spectrum of β-Mo_2_C was calculated using the initial cell suggested by Shi et al. [27]. The cell was optimised using the Broyden–Fletcher–Goldfarb–Shannon algorithm, and the generalised gradient approximation for exchange-correlation term according to Perdew–Burke–Ernzerhof implemented in the quantum ESPRESSO code [28] was used to perform first-principles calculations. The phonon spectrum was calculated based on the density functional perturbation theory. The details of the calculations can be found elsewhere [26]. The calculated structural parameters of β-Mo_2_C and α-MoC_0.5_ were *a* = 3.024 Å, *c* = 4.712 Å and *a* = 4.225 Å, respectively, in rather good agreement with the experimental values of *a* = 2.972 Å, *c* = 4.666 Å (the sample S2) and *a* = 4.227 Å (the sample S5). The calculated lattice parameter of cubic WC_0.5_ was 4.211 Å.

## 3. Results and Discussion

### 3.1. Phase Analysis of the nc-MoWC + α-C Nanocomposites

**Figure 1:**
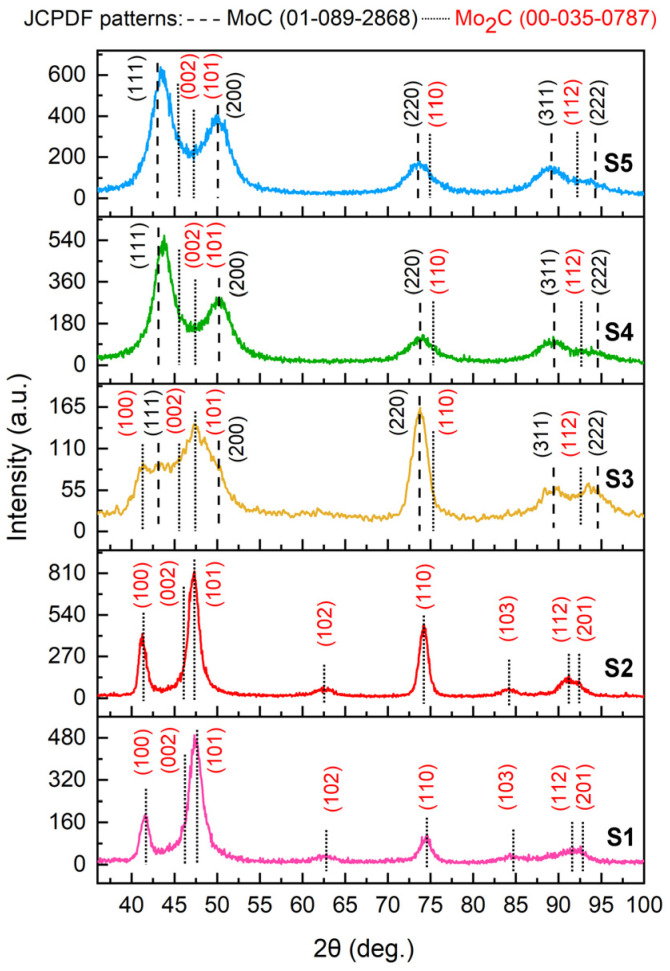
GI-XRD patterns of the as-deposited Mo-W-C films.

Figure 1 shows XRD patterns of five series of Mo-W-C films. It can be clearly seen that an increase in the power applied to the C target resulted in a change in phase composition. Samples could be divided into two groups. S1 and S2 carbides deposited at 15 W and 40 W consisted only of a hexagonal close-packed (*hcp*) β-Mo_2_C phase. However, applying a higher power (63 W, 84 W, and 110 W) drastically altered the structure of the Mo-W-C films.

Besides the β-Mo_2_C, they developed a face-centred cubic (*fcc*) α-MoC*_k_* phase. Diffraction peaks could be assigned to JCPDS pattern no. 00-035-0787 (P63/mmc, space group 194) and JCPDS pattern no. 01-089-2868 (Fm-3m, space group 225), *hcp* β-Mo_2_C and *fcc* α-MoC*_k_*, respectively.

The diffraction patterns of all carbides demonstrated the shift of peaks towards smaller angles compared to the reference JCPDS patterns. That was attributed to a decrease in the lattice parameters. The microstructural parameters based on the analysis of GI-XRD patterns are given in Table 2. 

The GI-XRD patterns of S1 and S2 films revealed (100), (101), (102), (110), (103), (112), and (201) diffraction lines assigned to the *hcp* molybdenum carbide. According to the first-principles density functional theory calculations, the β-Mo_2_C has the lowest formation energy within the Mo-C system. And when the carbon content increases, the *fcc* α-MoC*_k_* arises [29]. The absence of peaks attributed to the WC phase indicated that W atoms, having the same size as Mo atoms, substituted some amount of molybdenum atoms in the molybdenum carbide lattice, resulting in the formation of a solid solution β-(Mo + W)_2_C. Diffraction patterns of carbides deposited at 15 W and 40 W were characterised by a shift of diffraction lines to lower d-spacings compared to the reference JCPDS pattern of *hcp* Mo_2_C. However, the (100), (002), and (101) reflections were shifted more significantly, which could be caused by residual stresses. Change in the position of different XRD peaks of one phase can be explained by the need to balance stresses at the grain boundaries. The incorporation of W atoms into the molybdenum carbide crystalline structure deformed it, causing a change in the lattice parameters. The latter were decreased compared to those of the reference JCPDS β-Mo_2_C phase: *a*_(S1)_ = 2.958 Å, *c*_(S1)_ = 4.649 Å and *a*_(S2)_ = 2.972 Å, *c*_(S2)_ = 4.666 Å for coatings deposited at the power applied to the C target of 50 V and 100 V, respectively. Moreover, the higher X-ray peak intensities of the S2 film suggest a greater number of scattering atoms, indicating a higher degree of crystallinity.

Analysis of the XRD patterns of S3, S4, and S5 carbides revealed the double-phase structure. The Mo_2_C phase was represented by (100), (002), (101), and (112) diffraction lines, while other peaks were assigned to the (111), (200), (220), (311), and (222) reflections of the *fcc* α-MoC*_k_*. Similar to the above-described series of the Mo-W-C films, no peaks ascribed to the WC phase were observed. That indicated the formation of the substitution solid solution α(Mo + W)C*_k_*. However, the ratio between the two phases of molybdenum carbide varied depending on the power applied to the C target. Films of the S3 series deposited at 63 W still revealed a high volume of the β-Mo_2_C phase of about 47%, whereas there was approximately 53% of the α-MoC*_k_*. The XRD peaks were broader compared to the samples consisting only of the hexagonal phase, and a significant reduction in the mean crystallite size to 4.8 nm was observed. The lattice parameters were the following: *a*_Mo2C(S3)_ = 2.933 Å, *c*_Mo2C(S3)_ = 4.622 Å, and *a*_MoC(S3)_ = 4.217 Å. Higher C target power significantly increased the fraction of the *fcc* MoC, making it a dominant phase. S4 and S5 samples consisted of 28% (Mo_2_C)/72% (MoC*_x_*) and 29% (Mo_2_C)/71% (MoC*_k_*), respectively. The lattice parameters of the carbide deposited at 84 W were close to those of the coating obtained at 63 W: *a*_Mo2C(S4)_ = 2.928 Å, *c*_Mo2C(S4)_ = 4.619 Å, and *a*_MoC(S4)_ = 4.219 Å. Applying the highest power to the C target did not change the structure, but a slight increase in intensities of XRD peaks and lattice constants was detected: *a*_Mo2C(S5)_ = 2.941 Å, *c*_Mo2C(S5)_ = 4.634 Å, and *a*_MoC(S5)_ = 4.227 Å.

Therefore, an increase in the power applied to the carbon target led to a drastic change in the phase composition from a single-phase to a double-phase with an increase in the *fcc* α-MoC_k_ percentage from 0% to 72%. Based on Equations (1) and (2), the dimensions of the nano-grains were calculated. It was found that the deposited Mo-W-C films had a nanocomposite structure: nanograins of metal carbides with an average grain size within the range of 4–11 nm embedded into the amorphous C matrix.

### 3.2. Morphology and Chemical Composition of Nanocomposite Films

**Figure 2:**
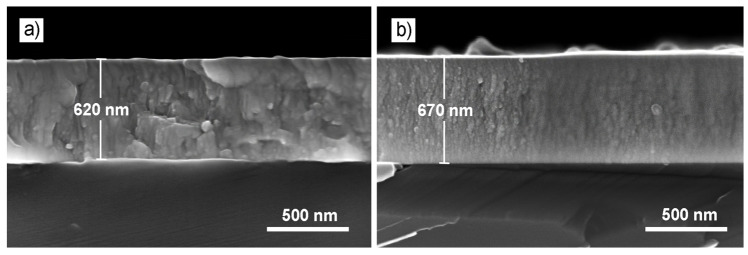
SEM cross-section images of the samples S1 (**a**) and S4 (**b**).

The typical SEM cross-section images of the Mo-W-C films on the example of S2 and S4 samples are provided in Figure 2a,b. The total thicknesses were in the range of 620–930 nm. The Mo-W-C films developed a dense homogeneous morphology. However, one can notice that the S4 film demonstrated a denser and more fine-grained microstructure compared to the S2, which was connected to their different phase compositions. The formation of two molybdenum carbide phases (*hcp* and *fcc*) embedded into the amorphous carbon resulted in the reduction in grains. The elemental composition determined by the EDS method is given in Table 3. The XPS analysis of the high-resolution spectra demonstrated that Mo-W-C films contained Mo-C, C-O, C-C, and C-O-C bonds, proving the formation of the nanocomposite structure with an amorphous carbon matrix. However, the W content was slightly lower compared to the EDS results.

**Figure 3:**
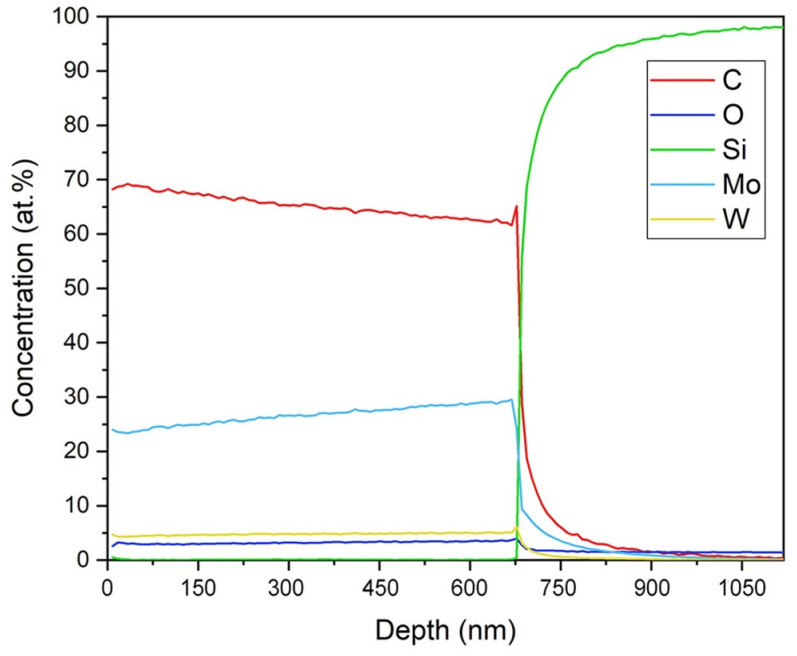
Typical SIMS profile of the Mo-W-C films (S4 sample).

Figure 3 shows the SIMS depth profile of the deposited Mo-W-C film of the S4 series. The distribution of ^12^C^+^, ^16^O^+^, ^94^Mo^+^, and ^182^W^+^ ion currents within the film was homogeneous. The stable deposition of the material created a uniform layer on the silicon substrate. There are no significant changes in the concentrations of individual components throughout the film thickness up to 670 nm. The analysis of oxygen distribution in the Mo-W-C film was complex since O_2_^+^ ions were used for the primary ion beam. Therefore, the oxygen signal during the Mo-W-C film sputtering was caused by the O_2_^+^ ions used for the element depth profiling. Moreover, the part of the oxygen plot concerning the Si substrate (above 670 nm) was burdened with a large error and did not describe the oxygen concentration.

**Figure 4:**
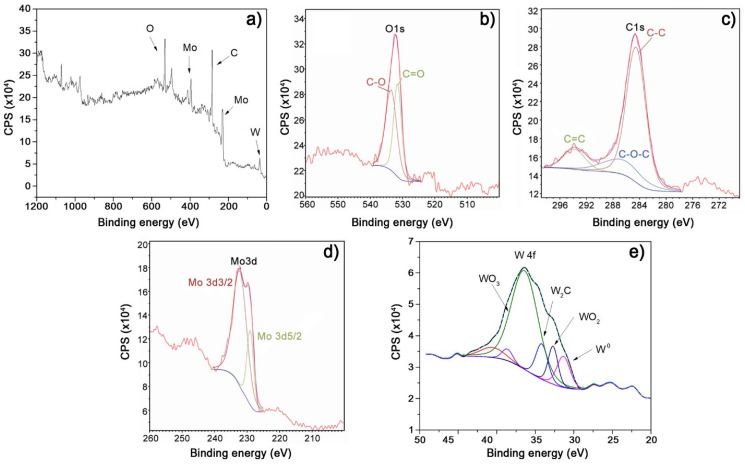
XPS survey spectrum (**a**) of Mo-W-C films (S4 sample) and high-resolution XPS spectra of O1s (**b**), C1s (**c**), Mo3d (**d**) and W 4f (**e**).

The elemental composition determined by the EDS method is given in Table 3. The XPS spectrum obtained after sputtering the surface layer for 30 min (Figure 4a) reveals the presence of Mo, W, C, and O elements in Mo-W-C films and their involvement in chemical bonding. Spectra demonstrated that films contained Mo-C, C-O, C-C, W-C, W-O, and C-O-C bonds (Figure 4b–d), proving the formation of the nanocomposite structure with an amorphous carbon matrix. High-resolution XPS analysis of the W 4f region (Figure 4e), obtained through deconvolution of the total spectrum, delineates various W bonds, including peaks corresponding to W_2_C, WO_3_ and WO_2_ oxides, and pure W with differing concentration ratios [30,31,32].

It is important to note that the results obtained via XPS differ from those obtained through XRD structural analysis (Figure 1) due to the disparate sensitivities of these methods. The detection limits of XRD are approximately 6–7%, whereas XPS boasts a significantly higher sensitivity with a detection limit of 1 monolayer, corresponding to 10^−3^ at.%. Consequently, the detection and precise quantification of pure W, W2C, and oxides, which may vary by units of atomic percentages or be present in very low quantities, pose a challenge to the XRD technique [33,34]. Analysis of XPS spectra reveals that carbon is bonded with molybdenum (α-MoC and β-Mo_2_C) and is present in the form of free (amorphous) carbon. This indicates the formation of Mo_2_C, MoC, and W_2_C grains, which are surrounded by a-C, C-O-C and a small fraction of WO_2_ and WO_3_ (spectrum W 4f). Therefore, the results of XRD analysis, complemented by the findings of XPS analysis, provide sufficient evidence to assert that through deposition, nanocomposite films containing nanoscale carbides within a matrix composed of amorphous carbon with a minor presence of oxide phases are created.

### 3.3. Analysis of the Influence of Fabrication Parameters of Nanocomposite Films on Their Structure

Based on the values presented in Table 2, including the phase composition of the carbides (Mo_2_ + W_2_)C and (Mo + W)C_(0.65≤*k*≤1)_, as well as Table 3—the concentration of Mo, W, and C elements, the carbon content of the carbides and its excess content were calculated. The results of the calculations are shown in Table 4.

The calculations were made based on the following assumptions:

It was assumed that the X-ray densities of both carbides are very close [35], amounting to 9.06 g/cm^3^ for Mo_2_C and 9.15 g/cm^3^ for MoC. The differences are only 1%. Therefore, the concentration of Mo atoms in both phases is practically the same, and its sum is equal to the value given in Table 3.

The XRD patterns showed that for samples 1 and 2, only the Mo_2_C phase was present. According to the spectra, it was determined that the tungsten atoms are in the crystal lattice, inserting the molybdenum atoms (solid equilibrium solution). Then, the tungsten atoms, as well as the molybdenum atoms, are incorporated into the carbide in the form of W_2_C.

It was taken into account that, according to the different authors, the α-MoC*_k_* phase found in samples S3–S5 is stable at 0.65 ≤ *k* ≤ 1.0 and is more energetically favourable than at *k* = 1.0 [26,35,36,37,38,39,40,41]. Calculations of phonon spectra of the cubic MoC phase revealed dynamical instability of the stoichiometric α-MoC. Moreover, in the NaCl-type transition metal carbides such as molybdenum carbide, it is rare to see all octahedral sites filled, and often, they have a broad single-phase region with high carbon substoichiometry [29]. Furthermore, it was shown that substituting Mo or C by a small amount (up to 5 at.%) of other transition metals stabilised the cubic δ-MoC*_k_* phase [42]. Considering this, in Table 4, the carbon content in carbides and excess carbon content are calculated for the extreme values of *k* equal to 0.65 and 1.0.

**Figure 5:**
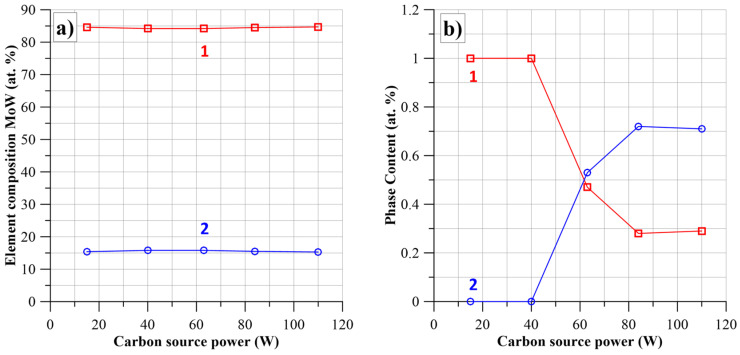
Elemental content of Mo—1, W—2 in MoWC nanocomposites depending on the strength of the carbon source (**a**), and dependencies of the content of the phases [(Mo_2*x*_ + W_2(1−*x*)_)C]*_y_*—1, and [(Mo*_x_* + W_(1−*x*)_)C*_k_*]_(1−*y*)_—2 (**b**).

The results of the carbide composition calculations and excess carbon content for these samples are given in Table 4. Figure 5a shows the dependence of Mo and W elemental content on the carbon atoms’ source power (Table 4). It shows that, on average, there is one tungsten atom for about 5.5 molybdenum atoms. The constancy of the metal element content is because the power of the source of metal atoms is practically constant (Table 1).

The presence of excess carbon indicates that the films produced are nanocomposites, where the matrix is composed of amorphous carbon with carbide nanograins in it. The chemical composition of the grown films was determined based on the data from Table 2 and Table 4. For samples S1 and S2, the chemical composition of the nanocomposite can be determined by the equation:

(4) $$nc-\left(Mo_{2x}+W_{2\left(1-x\right)}\right)C+\alpha -C_{z},$$

where *x*—content of Mo atoms; (1 − *x*)—content of W atoms, their sum is 1.00—Table 3; *z*—excess content of amorphous C atoms—Table 4.

As can be seen from the X-ray analysis, samples S3, S4, and S5 contain two types of carbide nanoparticles with basic compositions of (Mo_2_ + W_2_)C and (Mo + W)C_k_. It is evident from the X-ray diffraction patterns that these are also solid equilibrium solutions. In determining the composition of the carbides and the excess carbon content, it was taken into account that tungsten atoms can be present in the carbides in three ways. Firstly, all W atoms can be present in the carbide Mo_2_C nanograins, where they are found as W_2_C. Secondly, they are found in carbide MoC nanograins in the form of WC. Thirdly, in both types of nanograins. In Mo_2_C, nanograins are in the form of W_2_C, and MoC_k_ nanograins are in the form of WC_k_. It should be noted that the first and second cases are unlikely. In the third case, the content of tungsten atoms in each phase is close to proportional to the volume of the phases. Based on the above analysis of the nanograin composition, the content of carbon atoms included in the phases and present in excess form was calculated. Based on the calculation results presented in Table 4, a generalised formula for the composition of nanocomposites can be written:

(5) $$nc-\left[{\left(\left(Mo_{2x}+W_{2\left(1-x\right)}\right)C\right)}_{y}+{\left(\left(Mo_{x}+W_{\left(1-x\right)}\right)C_{k}\right)}_{\left(1-y\right)}\right]+\alpha -C_{z},$$

where *x*—the content of Mo atoms, (1 − *x*)—the content of W atoms, *y*—the content of (Mo_2*x*_ + W_(1−*x*)_)C phase, (1 − *y*)—the content of (Mo*_x_* + W_(1−*x*)_)C*_k_* phase, *z*—the content of excess carbon, 0.65 ≤ *k* ≤ 1.0. Values of the *x*, *y*, and *z* coefficients are given in Table 4.

From the results presented in Table 4, it can be concluded that increasing the power of the carbon atom source initially leads to an increase in the excess carbon content (samples S1, S2). In these samples, nanograins are present in the form of the (Mo_2_ + W_2_)C phase, and tungsten is found in the structure of molybdenum carbide in the form of inclusion atoms. Further increase in power causes the appearance of the (Mo + W)C*_k_* phase alongside the (Mo_2_ + W_2_)C phase. This requires an increase in the demand for carbon atoms that make up the carbides and, hence, a decrease in excess carbon content (sample S3). Further increase in the power of the carbon atom source leads to a decrease in the content of the (Mo_2_ + W_2_)C phase and an increase in the content of the (Mo + W)C*_k_* phase. At the same time, there is an increase in the excess carbon content (samples S4, S5).

Figure 5b shows the dependencies of the (Mo_2_ + W_2_)C and (Mo + W)C*_k_* phase content on the power of the carbon atom source. The most important conclusion drawn from the analysis of Figure 5b is the fact that there is a change in the phase composition of the nanocomposites when the power of the carbon atom source is increased at a constant power of the metal atom source. From Figure 5b, it can be seen that at low values of the power of the carbon atom source, the (Mo_2_ + W_2_)C phase accounts for 100% of the volume of the nano-grains. The increase in power leads to a decrease in the content of the (Mo_2_ + W_2_)C phase to about 47% of the volume and the appearance of the (Mo + W)C*_k_* phase. Further increase in power leads to a decrease in the content of the (Mo_2_ + W_2_)C phase to about (28–29)% and stabilisation of the (Mo + W)C*_k_* phase at about (71–72)%.

Such changes in the composition of metal carbide nanograins are due to the fact that as the power of the source increases, there is an increase in the ratio of carbon atoms to metal atoms. This favours the formation of ((Mo*_x_* + W_(1−*x*)_)C_k_)_(1−*y*)_ carbides, which contain twice as many carbon atoms per metal atom compared to carbides of ((Mo_2*x*_ + W_2(1−*x*)_)C)*_y_* composition. This means that by varying the power of the source of carbon atoms with a constant power of the source of Mo and W atoms, the phase composition of the carbide nanograins of these metals can be controlled.

### 3.4. Frequency–Temperature Measurements of the AC Properties of nc-MoWC + α-C Nanocomposites

#### 3.4.1. Hopping Conductivity by Electron Tunnelling

As is well known, nanocomposites often exhibit so-called hopping conductivity, which was initially observed in compensated semiconductors [43]. The mechanism is based on the quantum mechanical phenomenon of electron tunnelling between potential wells of nanometer dimensions, the distances between which are also nanometer. Analysis of the X-ray diffraction spectra, the results of which are shown in Table 2, showed that the films produced have a nano-grain structure. The nano-grains are located in a matrix consisting of excess amorphous carbon. This structure of the layers favours the occurrence of hopping conductivity.

In this paper, the analysis of the AC properties of the fabricated nanocomposites was based on the mechanism of hopping conductivity. Models describing this phenomenon were developed starting in the 1950s. Initially, research focused on DC conductivity. Subsequently, models were developed to describe alternating-current conductivity [44]. According to this model, the frequency dependence of the conductivity is described by the formula:

(6) $$\sigma \left(f\right)\sim f^{S},$$

where *S* = const ≤ 0.8.

In the paper [45], a model was proposed that simultaneously describes both the AC and DC conductivity of nanocomposites. It was initially applied to analyse the conductivity in highly defective GaAs. The model was then further developed in paper [46]. This paper considered the occurrence of a relaxation time for a tunnelling mechanism for the first time. Its occurrence is that an electron, after tunnelling from one well to another, cannot immediately perform another tunnelling. The electron, after the act of tunnelling, remains in the well for a time τ, called the relaxation time, and only after this time can it perform the next tunnelling [47]. The presence of a relaxation time makes the frequency factor in Equation (6) become a function of frequency [47]:

(7) $$\sigma \left(f\right)\sim f^{\alpha \left(f\right)}.$$

The value of α(*f*) is determined from the σ(*f*) waveforms by numerical differentiation:

(8) $$\sigma \left(f\right)=\frac{d\left[\log\sigma \left(f\right)\right]}{d\left(\log f\right)}.$$

**Figure 6:**
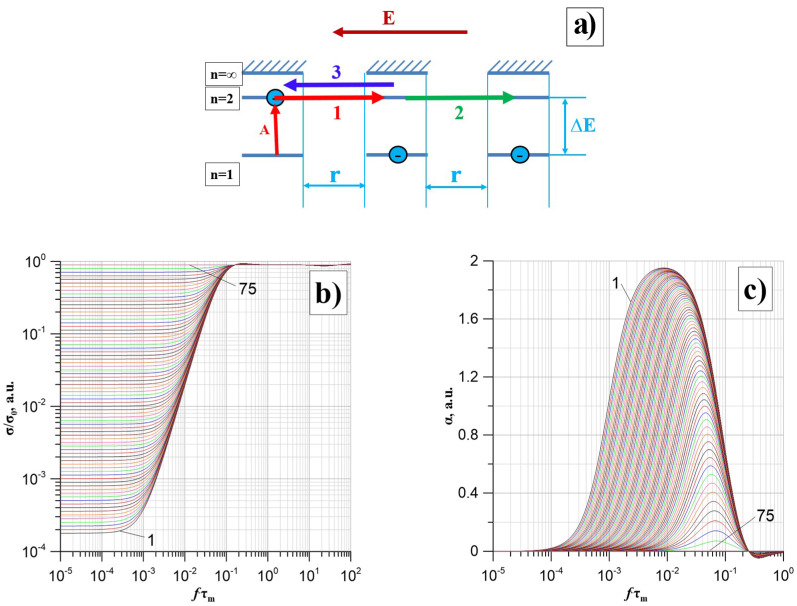
Tunnelling between potential wells in nanocomposites (**a**): A—thermal excitation of an electron, 1—tunnelling from the left well to the middle well, 2—tunnelling from the middle well to the right well, 3—return tunnelling from the middle well to the left well, ∆*E*—activation energy of tunnelling, *r*—distance between wells of potential. Dependence of σ(*f*)/σ_0_ (**b**) and α(*f*) (**c**) on the argument (*f*·*τ*_m_) for 75 probability values *p* from 10^−4^ (1) do 0.5 (75).

Electrons located in the potential well on the highest occupied state can participate in tunnelling (Figure 6a). Direct tunnelling to the same state that is occupied by an electron in an adjacent well is prohibited by the Pauli principle [48]. Tunnelling into a vacant state is possible. This means that the electron should be excited to the lowest unoccupied state before tunnelling. This is achieved by absorbing thermal energy quanta-phonons (Figure 6a). The difference in energy between the highest occupied and lowest unoccupied state is called the activation energy of thermally assisted tunnelling. In the case of conductivity measurements, this is the activation energy of conductivity.

Under the influence of an alternating electric field, a current of 1 (Figure 6a) is generated along the tunnelling path between the left and centre potential wells, the density of which is:

(9) $$j_{1}=\sigma_{0}E=\sigma_{0}E_{0}\sin\omega t,$$

where *E*—AC electric field strength, *E*_0_—electric field strength amplitude, *σ*_0_—conductivity, *ω*—circular frequency, *t*—time.

Tunnelling results in an electric dipole because the left well has lost an electron and has a positive charge. The middle well, on the other hand, has gained an extra electron and, therefore, has a negative charge. As a result of the dipole formation, additional polarisation of the nanocomposite occurs [49]. The electron, after tunnelling, remains in the well for a relaxation time *τ*. After the relaxation time, the electron can perform another tunnelling. This tunnelling can be performed by the electron in two ways. Firstly, the electron with probability *p* tunnels further from the middle well to the right well. This results in the formation of a second component of the current. This component is phase-shifted with respect to the external alternating electric field, and its value is:

(10) $$j_{2}=\sigma_{0}E_{0}p\sin\left[\omega \left(t-\tau \right)\right].$$

Secondly, the electron tunnels from the middle well to the left well with probability (1 − *p*). As a result of such tunnelling, a third component of the current density appears, which is phase-shifted concerning the alternating electric field—Figure 6a:

(11) $$j_{3}=\sigma_{0}E_{0}\left(1-p\right)\sin\left[\omega \left(t-\tau \right)\right].$$

Equations (9)–(11) allow the determination of the real current density component associated with the AC conductivity:

(12) $$j_{R}=\sigma_{0}E_{0}\left[1-\left(1-2p\right)\cos\left(\omega \tau \right)\right]\sin\left(\omega t\right).$$

From Equation (12), the *DC* conductivity (low-frequency) was determined:

(13) $$\sigma_{DC}=2p\sigma_{0}.$$

The high-frequency conductivity is, in this case:

(14) $$\sigma_{H}=\sigma_{0}.$$

In the intermediate frequency region, a frequency dependence of the current density is observed, described by Equation (7).

As established in the paper [47], the relaxation time value included in Equations (10)–(12) depends on the distance between the potential wells.

In nanocomposites, the potential wells are formed by the nanoparticles of the dispersed phase. Their distribution in the matrix is random or close to it. The random distribution of the wells results in a probability distribution of relaxation times. The distribution should be determined for positive values of relaxation times. Negative relaxation times mean that the electron will perform a return tunnelling from the middle well to the left well—before tunnelling from the left well to the middle well. As a distribution corresponding to these assumptions, the Landau probability distribution of the Moyal approximation can be used, among others [50]:

(15) $$F_{LM}\left(\tau \right)=\frac{1}{\sigma_{m}\sqrt{2\pi }}\exp\left(-\frac{\tau -\tau_{m}}{2\sigma_{m}}-\frac{1}{2}\exp\left(-\frac{\tau -\tau_{m}}{\sigma_{m}}\right)\right),$$

where *τ_m_*—the expected relaxation time value; *σ_m_*—standard deviation.

Taking into account the probability of relaxation times, Equation (12) for the density of the real AC component takes the form:

(16) $$j_{R}\left(\omega \right)=\sigma \left(f\right)E=\sigma_{0}E_{0}\sin\omega t\int_{\tau }F_{LM}\left(\tau \right)\left[1-\left(1-2p\right)\cos\omega \tau \right]d\tau .$$

The determination of the frequency dependence of the conductivity according to Equation (16) was performed by numerical methods for probability values *p* ranging from 10^−4^ to 0.5. The results of the calculations in the form of 75 dependence of *σ*(*f*)/*σ*_0_ on the argument (*f*·*τ_m_*) are shown in Figure 6b.

The figure shows that the conductivity is a constant value in the low and high-frequency areas. As *p* increases, the difference between the DC and high-frequency conductivity decreases. In the intermediate frequency region, an increase in conductivity is observed, Equation (7). The values of the coefficient α(*f*), Equation (7), were determined by numerical differentiation of the waveforms shown in Figure 6b. The results of the calculations for 75 values of *p* are shown in Figure 6c. The waveforms in Figure 6c illustrate the rate of change in conductivity with increasing frequency. It can be seen from Figure 6c that for small probability values of *p*, the maximum values of the frequency factor α_max_ are close to 2. As the probability value *p* increases, the maximum value α_max_ decreases, and the position of the maximum shifts to the higher frequency region. The value of the frequency at which the maximum occurs depends on the value of the relaxation time τ and, therefore, on the temperature. As the temperature increases, the value of the relaxation time decreases. This shifts the position of the αmax to the higher frequency region. In some nanocomposites, such as, for example, nc-Ti*_x_*Zr_1−_*_x_*C + α-C*_y_* [51], several types of potential wells may be present, differing in composition and structure. This results in the occurrence of several expectation values of relaxation times. In this case, the simultaneous occurrence of several maxima was observed on the frequency dependence α(*f*) [51]. It should be noted that the occurrence of several tunnelling mechanisms with different expectation values of relaxation times in some cases may not lead to the occurrence of a sufficient number of maxima on the α(*f*) relation. When the expectation values of relaxation times for different types of potential wells are close to each other, a single broad maximum or even a frequency range with an almost constant value of α(*f*) ≈ *const* may appear instead of a certain number of distinct maxima. Experimental verification of the model has been performed in a number of papers; see, for example [52,53].

#### 3.4.2. AC Conductivity Measurements

As mentioned above, the deposited nanocomposite films consist of an amorphous carbon matrix and nc-MoWC carbide nanograins embedded in the matrix. Increasing the power of the carbon atom magnetron source leads to changes in the structure and composition of the nanograins. At low powers (Table 4, films S1 and S2), the composition of the nanograins is described by Equation (4). With increasing power, two types of nano-grain compositions are observed in the films, described by Equation (5).

In the medium-power range, both components have almost equal volumes (Table 4, film S3). A further power increase results in an increase and then stabilisation of the volume of the second component, Equation (5) at (1 − *y*) ≈ (0.71–0.72) (layers S4 and S5, Table 4). Therefore, two nanocomposite films were selected for alternating current tests. The first was produced using the lowest carbon source power (Table 4, film S1). Its nanograin composition is described by Equation (4). The second layer tested was produced at a high carbon source power. It consists of two types of nanograins. Their composition is described by Equation (5) with a value of *y* ≈ 0.28 (Table 4, film S4).

As known, the basic AC parameters are conductivity and permittivity. Both of these parameters are part of Maxwell’s second equation [54] and describe the current flow in isotropic materials. A test stand described in publications [25] was used to measure these parameters. Measurements were made in the temperature range of 20–375 K. The measured resistance and capacitance values, electrode surface area, and layer thickness of 670 nm determined by SIMS and SEM cross-section methods were used to calculate conductivity and permittivity.

1. Conductivity of a single-phase film

Figure 6a shows the frequency dependencies of the conductivity of the S1 layer for selected measurement temperatures in the range of 20–375 K.

The frequency waveforms for temperatures above 300 K are similar to those obtained by computer simulation (Figure 6). The conductivity value is almost independent of frequency in the low-frequency region. In the higher frequency region, the conductivity starts to increase. The onset of the increase shifts with increasing temperature to the higher frequency region. This is associated with a decrease in the value of the relaxation time, described by the equation [55,56,57]:

(17) $$\tau =\tau_{0}\exp\left(\frac{\Delta W_{\tau }}{kT}\right),$$

where *τ*_0_—numerical value, Δ*W_τ_*—relaxation time activation energy.

**Figure 7:**
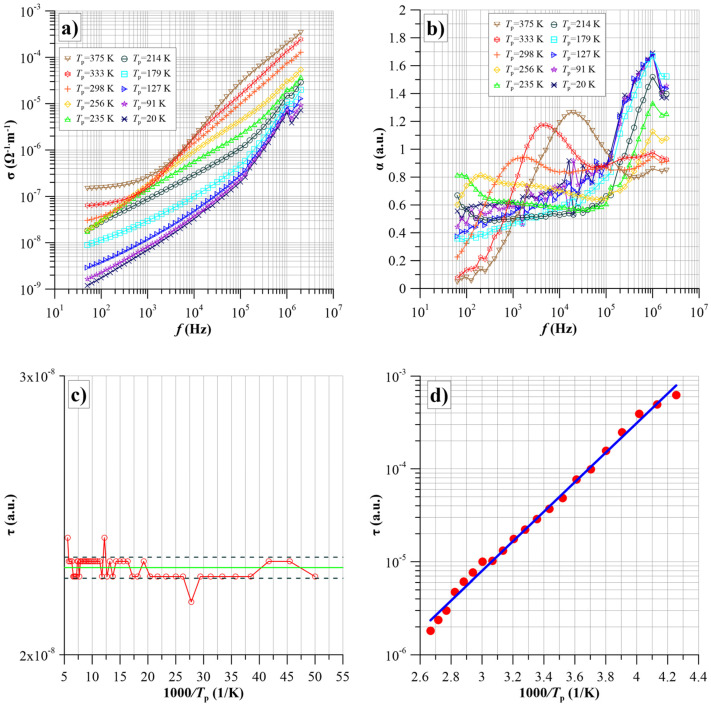
Frequency dependencies of conductivity—(**a**) of the frequency factor α(*f*)—(**b**) for selected measurement temperatures from 20 K to 375 K for the S1 sample. Arrhenius diagrams (**c**,**d**) for the S1 sample (green line shows the relaxation time for the high-frequency maximum).

For lower temperatures, only an increase in conductivity is observed on the frequency dependence. This is due to the fact that the steady-state transition is in the frequency region below the lower limit of the impedance meter range. It can be seen from Figure 7a that temperatures from 20 K to about 200 K have a weak effect on the frequency dependence of the conductivity. Up to a frequency of about 10^5^ Hz, a relatively slower-than-linear increase in conductivity is observed. Above this frequency, the increase in conductivity accelerates. Two factors simultaneously influence the position of the waveforms in relation to the logarithmic coordinates. The first is the temperature dependence of the conductivity, which shifts the displacement along the Y-axis. The second factor is the temperature dependence of the relaxation time, which shifts the waveform along the X-axis.

Figure 7b shows the frequency dependence of the α(*f*) factor for selected temperatures in the range 20–375 K, which shows the rate of increase in conductivity—Equation (7). For temperatures below 200 K in the frequency region up to about 10^5^ Hz, the α(*f*) values are close to each other and slowly increase starting from the lowest frequency. Above 10^5^ Hz, a rapid increase in α(*f*) begins until it reaches a maximum of α_max_ ≈ 1.7, occurring at a frequency of about 1 MHz. The position of this maximum and its amplitude do not depend on temperature. For temperature values above 200 K, the amplitude of the high-frequency maximum starts to decrease. At 375 K, this maximum practically disappears. A second low-frequency maximum appears in the low-frequency region at temperatures above 200 K. Its amplitude and the frequency of the position of the maximum increase with increasing temperature. Comparison of the α(*f*) waveforms at high temperatures with those obtained from the model (Figure 6c) shows that the α(*f*) values at low frequencies tend towards zero. This means that the low-frequency stage behaves according to the hopping conductivity model, considering the quantum mechanical electron tunnelling phenomenon. As shown above, the expected value of the relaxation time *τ_m_* for a given measurement temperature can be determined from the waveforms in Figure 7b. To do this, the values of α_max_ and *f*_max_ must be read off from the experimental waveforms. Then, from the family of waveforms shown in Figure 6, select the waveform that has a value at the maximum equal to the experimental value of α_max_ and read the value of the position of the maximum (*τ_m_ f*) for this waveform. The expected value of the relaxation time is then calculated from the equation:

(18) $$\tau_{m}\left(T\right)=\frac{\left(\tau_{m}f\right)}{f_{\max}},$$

Using this method, the relaxation times corresponding to the positions of the high-frequency and low-frequency maxima were determined from the results shown in Figure 7b. Arrhenius plots were chosen for the high-frequency maximum (Figure 7c) and the low-frequency maximum (Figure 7d).

Figure 7c shows that for the high-frequency maximum, tunnelling occurs with a very low, practically zero, activation energy. This situation is possible when there is a band of high-density energy states in the vicinity of the Fermi level, and the energy differences between adjacent states are less than 0.001 eV. This allows tunnelling with almost no thermal activation involved. This situation is similar to that observed in tunnelling diodes [58]. The value of the relaxation time for the high-frequency maximum is practically constant and is (2.27·10^−8^ ± 3.47·10^−10^) s for the whole temperature range. In the case of the low-frequency maximum, the Arrhenius diagram approximation yields a linear relationship, as evidenced by the unity-like value of the coefficient of determination for the linear approximation of the experimental values *R*^2^ ≈ 0.9944. On this basis, the activation energy of the relaxation time of the conductivity was determined to be Δ*W*_τ_ ≈ 0.316 eV.

2. Conductivity of a film with a two-phase composition

The S4 film, produced with a high-power source of carbon atoms, was chosen for the alternating current study. This led to a change in the structure of the carbide nanoparticles compared to the S1 film. Increasing the power led to a reduction in the nanoparticle content of the composition described by Equation (4) from 100% for film S1 to about 28% for film S4. The S4 film is dominated by nanoparticles of the composition described by the right-hand part of Equation (5), and their content is approximately 72%. Figure 6a shows the frequency dependence of the conductivity of the S4 film for selected measurement temperatures.

**Figure 8:**
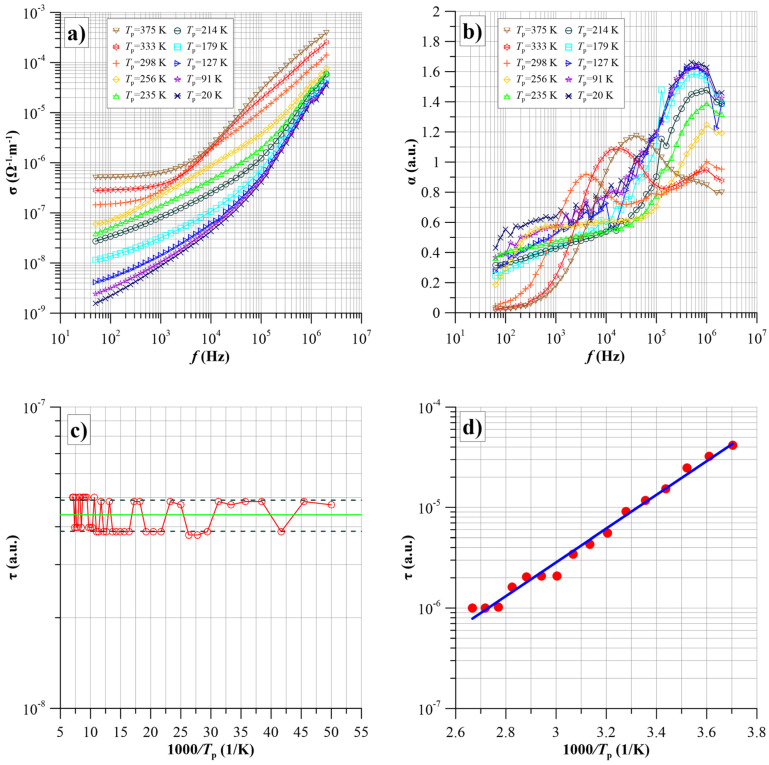
Frequency dependencies of conductivity—(**a**) of the frequency factor α(*f*)—(**b**) for selected measurement temperatures from 20 K to 375 K for the S4 sample. Arrhenius plots for the conductivity of the S4 sample: (**c**) for the high-frequency maximum (green line shows the relaxation time for the high-frequency maximum), (**d**) for the low-frequency maximum.

As for sample one, the measurement temperature range can conventionally be divided into two parts. The conductivity frequency waveforms are weakly temperature-dependent in the temperature area up to 200 K (Figure 7a). This is also clearly shown by the frequency factor waveforms α(*f*) (Figure 8b).

These waveforms slowly increase with increasing frequency up to approximately 2·10^4^ Hz. Once this value is overdone, the dependence of α(*f*) starts to increase rapidly until it reaches a maximum value of around 1.65. The frequency at which the maximum occurs is approximately 6·10^5^ Hz. Comparing the parameters of the maximum for film S4 with the corresponding ones for film S1, it can be seen that the α_max_ value has decreased slightly by about 0.05. In contrast, the *f*_max_ frequency decreased more markedly, from around 10^6^ Hz to around 6·10^5^ Hz. Furthermore, from a comparison of the waveforms in Figure 7b and Figure 8b, it can be seen that for the S4 film, the onset of the rise towards the high-frequency maximum starts at around 2·10^4^ Hz, that is, earlier than for the S1 film at about 10^5^ Hz. In the temperature area above 200 K, the highest frequency decreases and disappears at 375 K. At temperatures above 200 K, a low-frequency maximum appears. Its parameters also deviate from those determined for the S1 film. The amplitudes of these maxima for equal temperatures are slightly smaller than for the S1 film. In contrast, their frequencies of occurrence are shifted to the higher frequency area. The amplitudes and frequencies of occurrence of both maxima were determined from the experimental waveforms for film S4. Subsequently, the relaxation times for both maxima were determined in a manner analogous to that described for film S1. Arrhenius plots were determined from these, as shown in Figure 8c,d.

As with the S1 film, the relaxation time for the high-frequency maximum does not depend on temperature. However, the change in nanocomposite structure led to a twofold increase in its value, from (2.27·10^−8^ ± 3.47·10^−10^) s to (4.38·10^−8^ ± 5.18·10^−9^) s. This means that in both films, there is a band of electron states near the Fermi level, the energy distance between which is less than 0.001 eV. This allows tunnelling without the need for thermal activation.

For the S4 film, the activation energy of the low-frequency stage relaxation time is 0.333 eV. This value, within uncertainty limits, is equal to the relaxation time activation energies for the S1 film. The low-frequency stage is, presumably, associated with tunnelling from discrete states. This situation is shown in Figure 4a. The tunnelling electron is located in the potential well at the highest occupied level. For tunnelling to occur, it is thermally excited into the first unoccupied state. For this, thermal energy is needed, a value equal to the activation energy of the relaxation time, which is about (0.325 ± 0.009) eV for the investigated films.

### 3.5. Frequency–Temperature Dependence of Permittivity

**Figure 9:**
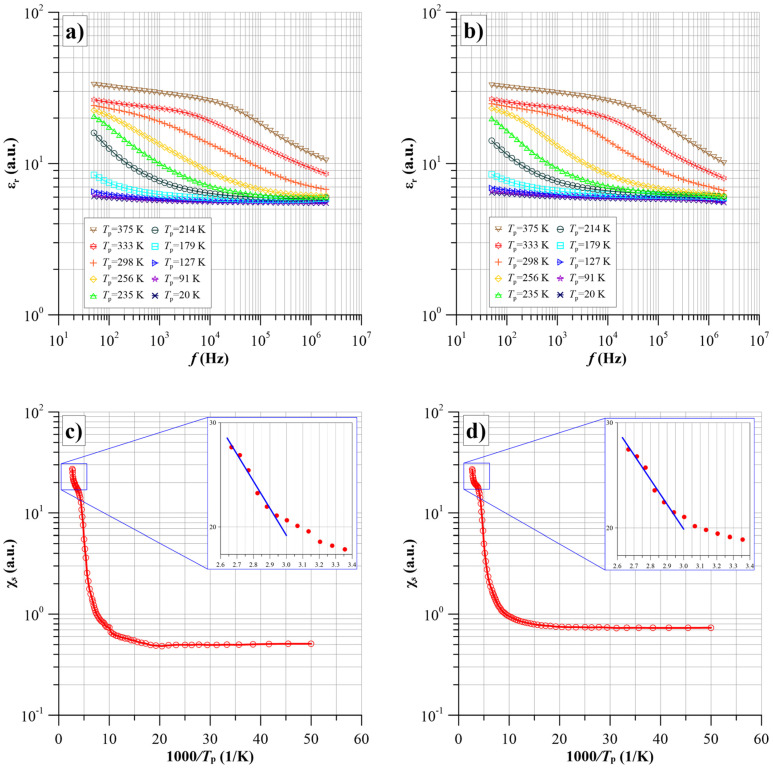
Frequency dependence of the permittivity for sample S1 (**a**) and S4 (**b**) for selected measurement temperatures in the range from 20 K to 375 K. Arrhenius plots for the static susceptibility of sample S1 (**c**) and S4 (**d**) (blue lines in the inserts represent linear approximations for waveform sections).

The frequency dependence of the permittivity for film S1 is shown in Figure 9a and for film S4 in Figure 9b. The analysis of the waveforms shows that in the low-temperature area, the permittivity does not depend on frequency and temperature. This is the so-called high-frequency permittivity, and its value is about 6. At temperatures above 150 K, an increase in permittivity is apparent in the low-frequency area.

As the frequency increases, the permittivity decreases and tends towards high frequency. Initially, the increase occurs at the lowest frequency. As the temperature rises, the waveforms move into the higher frequency area, and the permittivity values at 50 Hz increase. For temperatures above 200 K, an almost flat section appears at low frequencies. This indicates the presence of a static permittivity, the value of which increases with increasing temperature. This eliminates the possibility of atomic polarisation, which does not depend on temperature, and orientation polarisation, for which the static permittivity decreases with increasing temperature.

Such a situation is characteristic of static polarisation caused by the formation of dipoles due to tunnelling. A model of such permittivity has been developed in work [59]. The model shows that the static susceptibility, occurring at frequencies below the downslope section, is described by the formula:

(19) $$\chi_{s}=\varepsilon_{s}-1=\frac{NP(T)\tau e^{2}r^{2}}{\varepsilon_{0}kT},$$

where ε*_s_*—static permittivity; χ*_s_*—static dielectric susceptibility; *N*—concentration of potential wells; *P*(*T*)—the probability of electron tunnelling per unit time per unit volume; *τ*—relaxation time; *e*—charge of the electron; *k*—Boltzmann’s constant; *r*—the average distance between potential wells.

The value of the probability of electron tunnelling per unit time per unit volume *P*(*T*) is given by equation [39]:

(20) $$P(T)=P_{0}\exp\left(-\frac{2r}{R_{B}}-\frac{\Delta E}{kT}\right),$$

where *P*_0_—numerical factor; *R_B_*—radius of the electron’s location in the well of the potential, the so-called Bohr radius; Δ*E*—tunnelling activation energy.

The average distance between the potential wells, *r*, and their concentration *N* are related by the relation [44]:

(21) $$r\approx N^{-\frac{1}{3}}.$$

In paper [47], a formula for the relaxation time for tunnelling was derived:

(22) $$\tau =\tau_{0}\exp\left(\frac{\beta \cdot r}{R_{B}}\right)\cdot \exp\left(\frac{\Delta W_{\tau }}{kT}\right),$$

where *τ*—relaxation time, *τ_o_*—unspecified numerical coefficient, *β*—numerical coefficient, the value of which, according to [60], is *β* ≈ (1.75 ± 0.05), *r*—the distance over which the electron tunnels, *R_B_*—Bohr radius of the tunnelling electron, ∆*W_τ_*—activation energy of the relaxation time.

Substituting Equations (20) and (21) and the relaxation time Equation (22) into Equation (19) we obtain:

(23) $$\chi_{s}=\varepsilon_{s}-1=\frac{N^{\frac{1}{3}}P_{0}\tau_{0}e^{2}}{\varepsilon_{0}kT}\exp\left(-\frac{2}{R_{B}N^{\frac{1}{3}}}-\frac{\left(\Delta E-\Delta W_{\tau }\right)}{kT}\right).$$

The activation energy of electron tunnelling is greater than the activation energy of the dipole lifetime, Δ*E* > Δ*W*_τ_. This is due to the fact that electron return tunnelling (position 3 in Figure 6), which leads to the disappearance of the dipoles, takes place in the electric field of the dipole. Therefore, the activation energy of the dipole lifetime Δ*W*_τ_, compared to the tunnelling activation energy Δ*E*, is reduced by the value of the dipole potential energy, expressed in eV:

(24) $$U_{d}=\frac{e}{4\pi \varepsilon_{0}\varepsilon r},$$

where *e*—charge of the electron; *ε*_0_—dielectric permittivity of the vacuum; *ε*—relative permittivity of the material; *r*—average distance between potential wells.

Therefore, in Equation (23), the activation energy difference (Δ*E* − Δ*W_τ_*) can be written in the form:

(25) $$\Delta E-\Delta W_{\tau }=U_{d}=\frac{e}{4\pi \varepsilon_{0}\varepsilon r}.$$

From Equation (25), it follows that the static susceptibility should increase with temperature due to the increase in the number of tunnelling per unit time per unit volume, Equation (20).

From the waveforms shown in Figure 9, it is evident that additional thermally activated polarisation in the studied nanocomposites is caused by electron tunnelling between potential wells. From a comparison of the waveforms in Figure 9 with those for α(*f*), Figure 7b and Figure 8b, it is apparent that where a large high-frequency maximum of α(*f*) is evident at low temperatures, the susceptibility is low. An increase in susceptibility is observed at temperatures where a low-frequency maximum is present (Figure 7b and Figure 8b). From Equation (19), the susceptibility is proportional to the expectation value of the relaxation time. The relaxation time value for the high-frequency stage of conductivity is more than two orders lower than that for the low-frequency stage. Therefore, the susceptibility at the high-frequency stage should be about two orders lower than at the low-frequency stage. Figure 9c,d show Arrhenius plots for the susceptibility of the first and fourth samples at 50 Hz. For both samples, there is practically zero activation energy of susceptibility in the low-temperature area (large values of 1000/*T*). With increasing values of *T*, the susceptibility starts to grow. This section corresponds to the temperature range from 170 K to 250 K, where the steady-state transition (static susceptibility) does not yet occur. The increase in susceptibility in this range is associated with a shift of the waveforms into the higher frequency area caused by a decrease in relaxation time. With a further increase in temperature, a section of steady-state values (static susceptibility) appears on the susceptibility waveforms. From this section, the actual energy of susceptibility activation, determined by Equation (25), can be calculated. Enlarged Arrhenius plots for these sections are shown in the inset of Figure 9. From these plots, the values of the susceptibility activation energy were determined.

Based on the linear approximations for these waveform sections, shown in the insets, static susceptibility activation energies were determined, which is equal to *U_d_* dipole energies according to Equation (25). For the S1 film *U_d_*_1_ ≈ 0.074 eV, while for the S4 film *U_d_*_4_ ≈ 0.066 eV. Taking into account the uncertainty in the determination of the activation energy, it can be said that the *U*_d1_ and *U*_d4_ values are equal within the limits of uncertainty, with *U_d_* ≈ (0.07 ± 0.004) eV.

Using Equation (25), we can calculate the average distance between the potential wells produced by the carbide nanograins in the films produced:

(26) $$r=\frac{e}{4\pi \varepsilon_{0}\varepsilon U_{d}}.$$

The average distance between the carbide nanograins was calculated by substituting the values of high-frequency permittivity, static susceptibility activation energy, and other constants into Equation (26). This amounts to *r* ≈ (3.4 ± 0.2) nm. It should be emphasised that this value is an average value. In the nanocomposite films produced, there are distances between adjacent carbide nanograins, both smaller and larger than the average value.

### 3.6. Analysis of the Tribomechanical Parameters of Mo-W-C Nanolayers

#### 3.6.1. Microhardness of Nanocomposites

**Figure 10:**
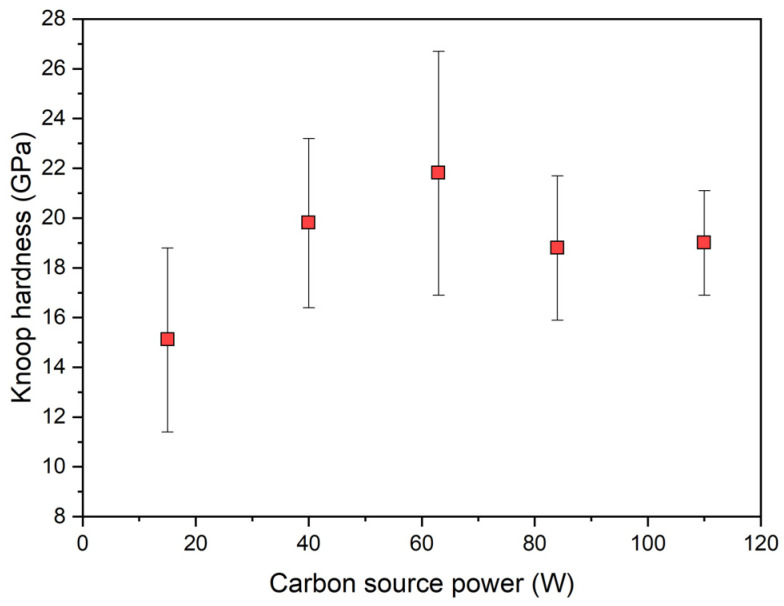
The Knoop hardness of Mo-W-C films depending on the power applied to the C target.

The Knoop hardness of the Mo-W-C films as a function of the carbon target power is depicted in Figure 10. It can be seen that the S3 sample with a ratio between hexagonal (Mo_2_ + W_2_)C and fcc (Mo + W)C_(0.65≤*k*≤1)_ phases of about 50%–50% demonstrated the highest hardness of about 21.8 ± 4.9 PGa. Meanwhile, Mo-W-C film deposited at the lowest power applied to the C target, which consisted of only (Mo + W)C_(0.65≤*k*≤1)_ phase, had the lowest hardness value of 15.1 ± 3.7 GPa. Moreover, S4 and S5, which developed similar *hcp*/*fcc* phase ratios, showed almost the same value, around 19 GPa. Therefore, the double-phase microstructure contributed to the increase in the hardness.

Mo-W-C coatings studied by Mandal [20] exhibited the best mechanical properties at the C/(Mo + W) of about 2, which was close to the carbon-to-metal ratios observed in present films with higher hardness. In addition, the calculated and experimental hardness values of β-Mo_2_C were only 11.38 GPa and 14.8 GPa, respectively [6]. Meanwhile, the first principles calculations by Liu et al. [39] predicted that the hardness of the cubic α−MoC is expected to be about 21.15 GPa, which surpasses that of the *hcp* phase. Close values were measured for nanocomposite thin films deposited at a higher power of the carbon target in this study. Furthermore, it should be noted that theoretical calculations demonstrated that doping molybdenum carbide with W resulted in enhanced mechanical modulus and elastic anisotropy; however, the effect on the hardness was negligible [61,62]. Hence, the enhancement of hardness was likely to be attributed to the nanocomposite nature of the deposited Mo-W-C films, i.e., a combination of hard carbide nanocrystallites with different strictures embedded into an amorphous carbon matrix [63].

#### 3.6.2. Tribological Properties

**Figure 11:**
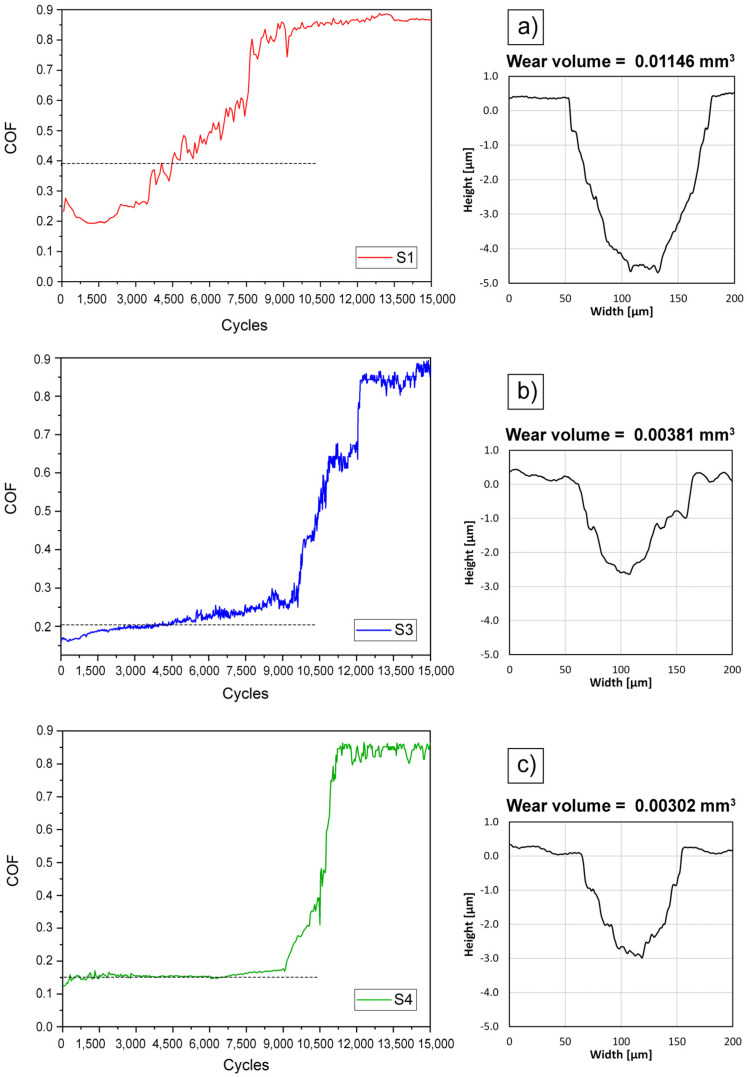
Evolution of the coefficient of friction depending on the number of cycles and 2D profiles of the wear tracks of S1 (**a**), S3 (**b**), and S4 (**c**) samples of Mo-W-C films after ball-on-disk at the load of 0.75 N. Horizontal dotted lines indicate the average values of the friction coefficients.

The wear resistance and friction performance of the Mo-W-C nanocomposites were analysed by ball-on-disc tests. Figure 11 shows the friction coefficient curves and 2D profiles of the wear tracks. Loads of 0.75 N were used because of the attempt to accurately determine the transition from a low coefficient of friction after the applied layer to a high coefficient of friction substrate. After layer failure, a rapid increase in the coefficient of friction was observed for all specimens tested, with an entry into a steady state corresponding to the substrate. Samples S1, S3, and S4 were chosen to compare the tribological performance of films with single- and double-phase structures. All films had a low coefficient of friction in the range of 0.15–0.39. Nanocrystalline carbide phases embedded in the amorphous carbon were found to have enhanced wear behaviour due to impeding the crack initiation and propagation. It can be clearly seen that a nanocomposite consisting of only the *hcp* phase demonstrated the worst wear resistance. Sample S1 had an average COF of about 0.39, and its wear scar was deep. Moreover, there was no obvious steady-state wear regime since COF progressively increased during sliding against the WC ball. However, a film composed of a combination of *fcc* and *hcp* carbides showed significantly lower friction coefficient and wear volume. The cubic molybdenum carbide demonstrated excellent wear resistance that could explain the superior tribological performance of S3 and S4 nanocomposites [64].

In the case of S3, it took about 10,000 cycles before the film wore off, but the wear scar was wider compared to the S4 nanocomposite. While the lowest volume loss and the COF of 0.15 were observed for sample S4. It remained intact up to 10,500 cycles, and even after total degradation, it kept the shallow wear track. The reason is the higher content of the cubic carbide in the S4 film at about 72% compared to the S3 with almost equal content of the fcc and hcp phases. It was found that tungsten oxides (W_87_O_13_, W_25_O_75_, WO_3_) and molybdenum oxygen-deficient Magnéli-phases (Mo_n_O_3n−1_) demonstrate low tribo-oxidation sensitivity and can serve as lubricants during friction enhancing the tribological properties [65,66]. Furthermore, the nanocomposite nature and formation of the double-phase structure with the prevalence of the fcc (Mo + W)C_(0.65≤k≤1)_ resulted in superior tribological performance. From Figure 11, it can be seen that the tests were carried out until the layers were completely rubbed off and recessed into the substrate. The wear of the layers, shown in Figure 11, was determined by indenting a ball equal to the thickness of the applied layers (Figure 5).

#### 3.6.3. Phonon Spectrum Calculations and Raman Spectroscopy

**Figure 12:**
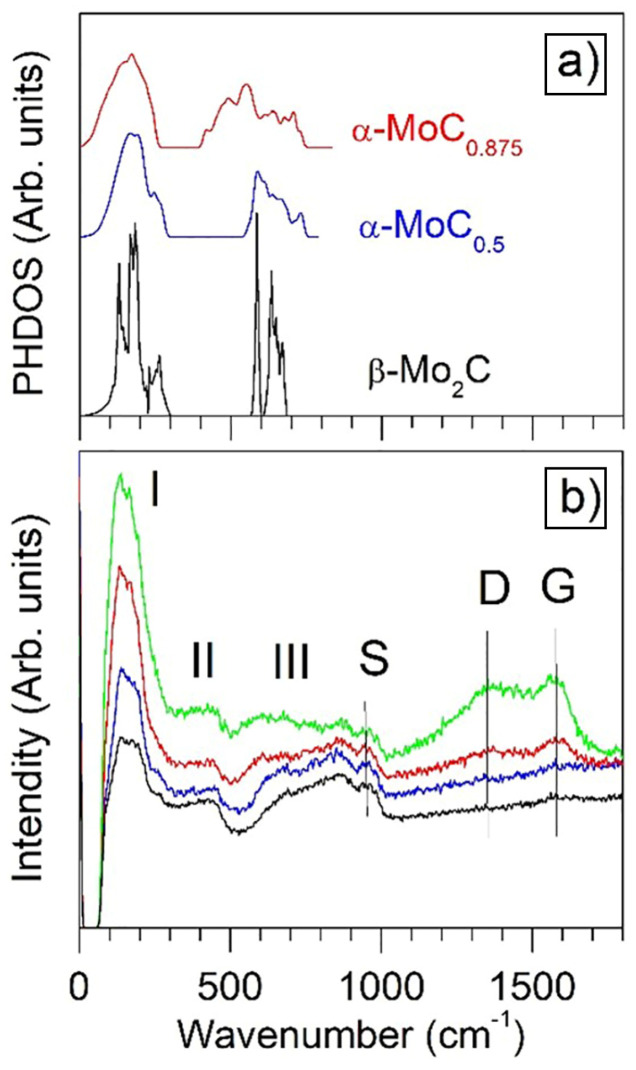
Calculated phonon density of states (PHDOS) of bulk α-MoC*_k_* [26] and β-Mo_2_C (**a**), and Raman spectra of the deposited S1 (black line), S2 (blue line), S3 (red line), and S4 (green line) coatings (**b**). Band and peak locations in the Raman spectra: 0–300 cm^−1^ (I), 300–500 cm^−1^ (II), 500–920 cm^−1^ (III), 960 cm^−1^ (S), 1356 cm^−1^ (D) and 1577 cm^−1^ (G). Adapted with permission from Ref. [26]. 2022, Elsevier.

The Raman spectra of the Mo-W-C films were analysed by comparing them to the phonon spectra of cubic α-MoC*_k_* (k = 0.5 and 0.875), hexagonal β-Mo_2_C phase, and cubic WC_0.5_ phases calculated based on the density functional theory. In Figure 12, we compare the calculated PHDOSs of molybdenum carbides with the Raman spectra of the deposited coatings. The Raman spectra are related mainly to molybdenum carbide since the molybdenum content in the films is much higher than the tungsten content. Furthermore, we found that the acoustics and optical vibration regions of cubic WC_0.5_ of 0–228 cm^−1^ and 540–773 cm^−1^, respectively, are close to those of α-MoC_0.5_ (cf. Figure 12) [67]. Figure 12a shows that the optical brunch in the PHDOSs of α-MoC*_k_* narrows with decreasing *k*.

Several bands and peaks can be distinguished in the Raman spectra: 0–300 cm^−1^ (band I), 300–500 cm^−1^ (band II), 500–920 cm^−1^ (band III), 960 cm^−1^ (peak S), 1356 cm^−1^ (peak D) and 1577 cm^−1^ (peak G). Comparing the Raman spectra with the calculated phonon spectra of the MoC*_k_* phases revealed in the films, we conclude that band I originated due to acoustic vibrations. Band II is not shown in the PHDOSs. This band is related to two-phonon scattering by anomalous phonons in the NaCl-type transition metal compounds [68]. The broad band III is supposed to be due to the optical vibrations (cf. Figure 12a). This band is much wider than the range of the optical vibrations, which can be related to the real randomised structure of the α-MoC*_k_* and β-Mo_2_C phases. Furthermore, oxygen can contribute to the high-energy region of this band. The second-order silicon peak S in the experimental spectra points to the transparency of the films [69]. The Raman bands at 470 and 960 cm^−1^ could indicate an appearance of the MoO*_x_* phase [70,71], whereas the band at 960 cm^−1^ could also be related to Mo hydration after ambient air storage [72]. Two prominent peaks, D and G, indicate the presence of the amorphous carbon phase in the coatings. The intensity of these peaks increases with the discharge power at the graphite target. This fact can be used for an explanation of the strength properties of the coatings. Obviously, there is an optimal thickness of the amorphous grain boundaries that promotes the strengthening of the coatings. We suppose that such an optimum structure is reached for sample S3, which has the maximal hardness. Thus, based on the discovered findings, it can be stated that applying high powers to the C target results in the formation of the nanocomposite structure represented by α-(Mo + W)C_(0.65≤*k*≤1)_ and β-(Mo_2_ + W_2_)C phases embedded into the amorphous carbon matrix.

## 4. Conclusions

In this study, five composite Mo-W-C films were produced using dual-source magnetron sputtering. During sample preparation, the power of the C source was gradually increased from 15 W to 110 W.

According to the analysis of XRD and Raman spectra, it was established that films consisted of metal carbide nanograins embedded in a matrix of amorphous carbon. The most significant finding of the stoichiometry study is that there were changes in the phase composition of nanocomposites when the power of the carbon source was increased. W atoms entered the composition of nanograins as substitutional atoms. At low power values (15 W and 40 W), 100% of the nanograin volume comprised the (Mo_2_ + W_2_)C phase. An increase in power resulted in a decrease in the content of the (Mo_2_ + W_2_)C phase and the appearance of the (Mo + W)C_(0.65≤*k*≤1)_ phase. A further increase in power led to a stabilisation of the *fcc* α-(Mo + W)C_(0.65≤*k*≤1)_ phase at around (71–72)%. This means that by varying the power of the source of carbon atoms, with a constant source power of Mo and W atoms, the phase composition of metal carbide nanograins can be controlled.

Measurements of electrical properties of two different films with nanograin compositions of (Mo_2_ + W_2_)C (100%) (S1) and (Mo_2_ + W_2_)C (28%) + (Mo + W)C_(0.65≤*k*≤1)_ (72%) (S4) demonstrated that the nanograin structure favoured the occurrence of hopping conductivity. The value of frequency factor α(*f*) showed the rate of the conductivity change with increasing frequency in a double-logarithmic coordinate system. For the S1 film with (Mo_2_ + W_2_)C composition, there was a high-frequency stage of increase in conductivity in the low-temperature region, which corresponded to a maximum α(*f*) at a frequency of about 1 MHz. The value at the maximum α_max_ ≈ 1.7 and the expected value of the relaxation time τ_m_ ≈ 2.27·10^−8^ s did not depend on temperature. The expectation value of the relaxation time decreases with temperature. The temperature dependence of the relaxation time was determined, and the activation energy of the relaxation time Δ*W*τ_1_ ≈ 0.316 eV was calculated.

For the S4 film with a nanograin composition of [(Mo_2_ + W_2_)C]_0.28_ and [MoWC*_k_*]_0.72_, similar conductivity and α(*f*) curves were observed as for the first sample. The change in the film composition led to some quantitative changes in the parameters describing the dependencies. For the high-frequency stage, the position of the maximum decreased to a frequency of 6·10^5^ Hz, and the value at the maximum of α_max_ decreased to 1.6. The expected value of the relaxation time increased to 4.38·10^−8^ s. These parameters did not depend on temperature. The activation energy of the relaxation time for the low-frequency stage decreased to Δ*W*τ_4_ ≈ 0.333 eV.

It has been established that the permittivity in the low-temperature region did not depend on frequency and temperature. The presence of a static permittivity was detected, the value of which increased with elevating the temperature. This means that there is an additional thermally activated polarisation in the nanocomposites studied caused by the formation of dipoles following the tunnelling of an electron from one neutral potential well to an adjacent one. For samples S1 and S4, the values of the potential energy of the dipoles were equal within the uncertainties, with an average value of (0.07 ± 0.004) eV. On this basis, the average distance between the carbide nanograins (the distance over which the electrons tunnel) was found to be (3.4 ± 0.2) nm. It should be emphasised that this value is an average value. In the synthesised nanocomposites, there are distances between adjacent carbide nanograins, both smaller and larger than the average value.

Furthermore, it was found that applying a low carbon source power of 15 W during the deposition process of Mo-W-C films resulted in the lowest Knoop hardness value of 15.1 ± 3.7 GPa. However, nanocomposites synthesised at a C power greater than 40 W demonstrated higher hardness with a maximum value of 21.8 ± 4.9 GPa for the sample with nanograins composed of hexagonal β-(Mo_2_ + W_2_)C and *fcc* α-(Mo + W)C_(0.65≤*k*≤1)_ phases. Thus, the double-phase microstructure contributed to the increase in the hardness. Tribological tests demonstrated that Mo-W-C films with a double-phase structure also had superior tribological performance. Particularly, the Mo-W-C film with the highest volume of the *fcc* α-(Mo + W)C_(0.65≤*k*≤1)_ phase of about 72% exhibited the lowest volume loss and COF of 0.15.

## Figures and Tables

**Table 1:** Deposition parameters of Mo-W-C films.

Sample	*T*_S_ (°C)	*U* (V)	*F*_Ar_ (sccm)	*p* (Pa)	Target Mo-W			Target C			Deposition Time (min)
					U (V)	I (mA)	P_Mo-W_ (W)	U (V)	I (mA)	P_C_ (W)	
S1	400	−50	58	0.2	310	200	62	300	50	15	60
S2	400	−50	58	0.2	340	200	68	400	100	40	60
S3	400	−50	58	0.2	340	200	68	420	150	63	60
S4	400	−50	58	0.2	330	200	66	420	200	84	60
S5	400	−50	58	0.2	330	200	66	440	250	110	60

**Table 2:** Microstructure parameters of the Mo-W-C films deposited at different power supplied to the C target: lattice parameters and volume fractions of phases identified using GI-XRD patterns.

Sample	Lattice Parameters (Å)			Phase Percentage (%)	
	β-Mo_2_C		α-MoC*_k_*		
	*a*	*c*	*a*	β-Mo_2_C	α-MoC*_k_*
S1	2.958	4.649	−	100	−
S2	2.972	4.666	−	100	−
S3	2.933	4.622	4.217	47	53
S4	2.928	4.619	4.219	28	72
S5	2.941	4.634	4.227	29	71

**Table 3:** Chemical composition of the Mo-W-C films obtained by the EDS method.

Sample	Elemental Composition (at.%)		
	Mo	W	C
S1	37.9	6.9	55.2
S2	31.4	5.9	62.7
S3	33.6	6.3	60.1
S4	26.5	4.8	68.7
S5	27.5	4.9	67.6

**Table 4:** Chemical composition of MoC coatings obtained by the EDS method.

Sample	Elemental Composition MoW		Structure of Molybdenum Carbide		C in Nanocomposite		
	Mo, a.u. (x)	W, a.u. (1 − x)	(Mo_2_ + W_2_)C, a.u. (*y*)	(Mo + W)C*_k_*_(0.65≤*k*≤1)_ a.u. (1 − *y*)	C in Carbides a.u.	Redundant C, a.u. (*z*)	Total C a.u.
S1	0.846	0.154	1	0	0.5	0.73	1.23
S2	0.842	0.158	1	0	0.5	1.18	1.68
S3	0.842	0.158	0.47	0.53	0.67 ± 0.09	0.81 ≤ *z* ≤ 0.99	1.57
S4	0.845	0.155	0.28	0.72	0.735 ± 0.125	1.50 ≤ *z* ≤ 1.75	2.36
S5	0.847	0.153	0.29	0.71	0.725 ± 0.125	1.32 ≤ *z* ≤ 1.57	2.17
Average	0.8444 ± 0.0022	0.1556 ± 0.0022					

## Data Availability

Data can be available upon request from the authors.
